# The Association Between Female Genital Schistosomiasis and Other Infections of the Lower Genital Tract in Adolescent Girls and Young Women: A Cross-Sectional Study in South Africa

**DOI:** 10.1097/LGT.0000000000000756

**Published:** 2023-06-28

**Authors:** Jilna Dilip Shukla, Elisabeth Kleppa, Sigve Holmen, Patricia D. Ndhlovu, Andile Mtshali, Motshedisi Sebitloane, Birgitte Jyding Vennervald, Svein Gunnar Gundersen, Myra Taylor, Eyrun Floerecke Kjetland

**Affiliations:** 1 Norwegian Centre for Imported and Tropical Diseases, Department of Infectious Diseases Ullevaal, Oslo University Hospital, Oslo, Norway; 2 Faculty of Medicine, University of Oslo, Oslo, Norway; 3 Imperial College London, Claybrook Centre, London, United Kingdom; 4 Department of Infection Prevention and Control, School of Laboratory Medicine and Medical Sciences, Nelson R Mandela School of Medicine, University of KwaZulu-Natal, Durban, South Africa; 5 Discipline of Gynaecology, Nelson R Mandela School of Medicine, College of Health Sciences, University of KwaZulu-Natal, Durban, South Africa; 6 Section for Parasitology and Aquatic Pathobiology, Faculty of Health and Medical Sciences, University of Copenhagen, Copenhagen, Denmark; 7 Department for Global Development and Planning, University of Agder, Kristiansand, Norway; 8 Discipline of Public Health Medicine, Nelson R Mandela School of Medicine, College of Health Sciences, University of KwaZulu-Natal, Durban, South Africa; 9 Department of Global Health, Oslo University Hospital, Norway

**Keywords:** Africa, *Chlamydia trachomatis*, human papillomavirus, parasitic infection, female genital schistosomiasis, female

## Abstract

This study aimed to explore the relationship between female genital schistosomiasis (FGS), sexually transmitted infections, bacterial vaginosis, and yeast among young women living in *Schistosoma haematobium*-endemic areas.

**Methods:**

In a cross-sectional study of young women, sexually active, aged 16 to 22 years in rural KwaZulu-Natal, South Africa, in 32 randomly selected rural schools in schistosomiasis-endemic areas, the authors performed gynecological and laboratory investigations, diagnosed FGS and other infections, and did face-to-face interviews.

**Results:**

Female genital schistosomiasis was the second most prevalent current genital infection (23%), significantly more common in those who had urinary schistosomiasis (35%), compared with those without (19%, *p* < .001). In the FGS-positive group, 35% had human papillomavirus compared with 24% in the FGS-negative group (*p* = .010). In the FGS-positive group, 37% were seropositive for herpes simplex virus infection, compared with 30% in the FGS-negative group (*p* = .079). There were significantly fewer chlamydia infections among women with FGS (20%, *p* = .018) compared with those who did not have FGS (28%).

**Conclusions:**

Female genital schistosomiasis was the second most common genital infection after herpes simplex virus. Human papillomavirus infection was significantly associated with FGS, but *Chlamydia* was negatively associated with FGS. Women with FGS may have had more frequent contact with the health system for genital discharge. The results show the importance of the inclusion of FGS in the national management protocols for genital infections in areas endemic for *S. haematobium* and highlight a more comprehensive approach to diagnosis and genital disease management.

Female genital schistosomiasis (FGS) was first described in 1899 in Egypt but was largely only reported as incidental findings till the 1990s.^1^ Female genital schistosomiasis has been found to be almost as common as urinary schistosomiasis within the female populations of *Schistosoma haematobium*-endemic areas.^2–4^ Furthermore, in some schistosomiasis-endemic communities, it may be the most common gynecological morbidity.^4^ Despite its prevalence, World Health Organization (WHO) data for 2017 show that only 46.3% of the 220.8 million people requiring treatment for schistosomiasis have been reached.^5^

Female genital schistosomiasis is a poverty-related neglected tropical disease caused by the *S. haematobium* parasite, which is transmitted by contact with infested fresh water.^4^ It is known to be endemic in areas of sub-Saharan Africa where lakes, rivers, and dams are used for domestic and recreational purposes.^6^ Female genital schistosomiasis may cause bloodstained and foul-smelling discharge, dyspareunia, contact bleeding, burning sensations in the genitals, decreased fertility, spontaneous abortions, and increased susceptibility to HIV and human papillomavirus (HPV), as well as possibly cervical cancer.^4,7,8^ The *S. haematobium* parasites inhabit blood vessels surrounding the female genital tract and the urinary bladder.^4^ There they lay eggs, which are then propagated toward the adjacent organs, creating the aforementioned morbidities. The morbidity seen in FGS is attributed to chronic inflammation in the cervical and vaginal mucosa, and lesions involve both dead and viable parasite eggs.^9^

Female genital schistosomiasis is a differential diagnosis to some sexually transmitted infections (STIs), causing vaginal discharge and ulcers.^10^ Furthermore, it has also been hypothesized that FGS may make women susceptible to HPV infection and other STIs.^11,12^

In this study, we aim to explore the relationship between lower genital tract FGS and reproductive tract infections in an adolescent female population and establish the relative prevalences of these infections in a rural *S. haematobium*-endemic area.

## METHODS

The study was approved by the Biomedical Research Ethics Administration (BREC), University of KwaZulu-Natal, South Africa (Ref BF09/07) and the Department of Health, Pietermaritzburg, South Africa (Ref HRKM010–08). Ethical clearance was also granted from the regional committee of Medical and Health Research Ethics (REC), South Eastern Norway (Ref 469-07066a1.2007.535) and renewed in 2011. Written informed consent was obtained from all subjects, a legal surrogate, and the parents or legal guardians for minor subjects. To maintain confidentiality, legal guardians and parents were not given information acquired from the young women during the consent procedure or investigations. Permissions were also granted by the Departments of Health and Education, KwaZulu-Natal. The STI and FGS testing and results were described in the consent form. At the end of the questionnaire, the participants were asked if, and how, they wished to be contacted, should tests show disease for which they needed treatment or referral. All participants with positive laboratory results were approached and were either given treatment by a registered nurse (tablets) or offered transport to a local clinic for management.

## Study Design

It has been shown that schistosomiasis is endemic in the lower altitudes KwaZulu-Natal Province of South Africa.^13^ Therefore, randomly selected rural secondary schools below the altitude of 400 m and with a urinary schistosomiasis prevalence greater than 20% were invited to take part in the study. For practical purposes and due to financial constraints, we only invited schools with 300 or more pupils. To reach as many positive cases as possible, schools were excluded if they had been classified as urban (and therefore less likely to be using infested water sources) by the Department of Education. From 2011 to 2013, nested in a prospective study on the prevention of FGS, the study included sexually active, school-attending women aged 16–22 years, usually in grades 10–12, who consented to a gynecological examination and were willing to be tested for STIs.^14^

## CLINICAL EXAMINATION AND QUESTIONNAIRE

Participants underwent a gynecological examination performed by female doctors trained by the same expert in recognizing FGS on the cervix and vaginal walls. Clinical manifestations of FGS were documented by photocolposcopy (Olympus OCS 500 [Olympus, Tokyo, Japan] colposcope with a mounted Olympus E 420 [Olympus, Tokyo, Japan] 10-MP single lens reflex device, or a Leisegang colposcope [Leisegang, Berlin, Germany] with a Canon EOS 40D [Canon, Tokyo, Japan] 10-MP single lens reflex).^15^ In average biweekly, an FGS expert would investigate patients together with the clinician to ascertain uniform diagnosis; furthermore, images were reviewed as quality control by 1 expert (E.F.K.). Grainy sandy patches were defined as lesions with grains measuring approximately 0.05 × 0.2 mm and shaped like minuscule rice grains, deeply or superficially situated within the mucosa.^10^ Homogenous yellow patches were defined as yellow areas with no distinct grains. The chronological aspects of FGS have not yet been determined, although 2 recent articles suggest that homogenous yellow patches seem to represent old lesions, whereas grainy sandy patches and rubbery papules represent more recent deposition of *S. haematobium* ova in the tissues.^16,17^ Rubbery papules were defined as beige to yellow papules with a firm (rubbery) protrusion into the intravaginal lumen. All the FGS lesions may be accompanied by characteristic abnormal blood vessels seen as pathological convoluted, uneven calibered, reticular, circular, branched, or corkscrew patterns.^18^ Patients were only investigated by 1 clinician.

Trained female staff interviewed study participants in isiZulu (the local language) using a questionnaire that covered demographic information, current reproductive health complaints, obstetric history, sexual behavior, water contact, and their approaches to the health services for genital symptoms. Those who said they had not commenced sexual activity were not invited for clinical examination.

## LABORATORY TESTING

For the diagnosis of urinary schistosomiasis, a urine sample was collected between 10 a.m. and 2 p.m., which is the peak daily egg excretion time.^19^ Urine was centrifuged, the sediment deposited on 2 slides, and read by 2 independent laboratory technicians.

Urine was considered schistosomiasis-positive if at least 1 *S. haematobium* ovum was seen under microscopy. Samples of cervicovaginal lavage were collected by spraying 10 mL of saline on the cervical surface and withdrawing it back into the syringe. Herpes simplex virus type 2 (HSV-2) antibodies were detected in serum using ELISA Ridascreen HSV-2 IgG (Davies Diagnostics, Randburg, South Africa) and in ELISA HerpeSelect, IgG assay (Focus Diagnostics, Germany). Herpes simplex (Cape Town, South Africa) virus type 2 polymerase chain reaction (PCR) test was only done in a subsample of participants due to financial and practical constraints. Likewise, HPV was done in a subsample and detected by GP5+/6+ HPV PCR test, followed by an enzyme immunoassay method using a cocktail mix of high-risk HPV (hr-HPV) probes (Whitehead Scientific, Cape Town, South Africa).^20^ For genotyping, individual probes were used. Individual probes for the most common hr-HPV genotypes in cervical cancer were selected: hr-HPV types 16, 18, 26, 31, 33, 35, 39, 45, 51, 52, 53, 56, 58, 59, 66, 68, 73. and 82. Fourteen of these were identified individually from specimens using EIA test, and HPV 26 and 53 as well as HPV 73 and 82 were tested in combination. The cut-off value for hr-HPV genotype was calculated following the formula of the mean optical density plus 3 times the standard deviation (SD) of all samples in the plate. After exclusion of outliers, the final mean + 3SD was recorded and used as the cut-off. This genotyping assay precludes detection of previously unknown genotypes. Cervical swabs were analyzed using ProbeTec CT/GC and AC strand displacement PCR assay for *Chlamydia trachomatis* and *Neisseria gonorrhea* (Becton Dickinson Microbiology Systems, Franklin Lakes, NJ).^21^ In-house validated PCR assays were done using the primer set SarR and SarF specific for *Trichomonas vaginalis* and HSV in the Laboratory of Infection, Prevention, and Control at the University of KwaZulu-Natal, Durban, South Africa.^22^ Syphilis was detected in thawed serum samples using Macro Vue test 110/112 (BD, Becton Dickinson Microbiology Systems) for a rapid plasma reagin test and Immutrep for *Treponema pallidum* hemagglutination assay (Omega Diagnostics Group PLC, Alva, United Kingdom). The Nugent scoring criteria was used for the diagnosis of bacterial vaginosis. Candidiasis was diagnosed using the Gram staining technique under light microscopy and severity scored (1–5) in accordance to the number of spores seen.^23^

## STATISTICAL ANALYSIS

To be able to detect a 10% difference in an STI's prevalence between the FGS-positive and negative groups with a confidence level of 0.05 and power of 0.8, sample size calculations indicated that we would need 200 FGS-positive patients and 600 FGS-negative patients. Chi-square or Fisher exact tests were used to evaluate the hypothesis. Age was included in the multivariable logistic regression regardless of significance level. Other variables were included if the association between the sandy patches and the STIs had a *p* value lower than 0.2. Comparison of mean age between the FGS-positive and negative groups was done using the Student *t* test. An α level of 0.05 was used for all statistical tests. Analyses were performed using IBM SPSS Statistics Version 25 (IBM Corp, Armonk, NY).

## RESULTS

**FIGURE 1:**
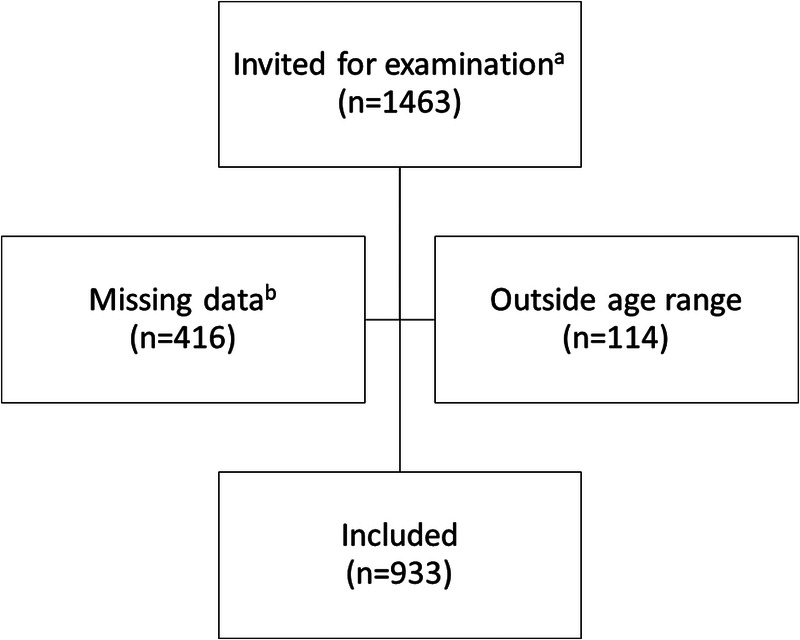
Flow chart of participants in the study. ^a^Female pupils in schools with more than 20% urinary schistosomiasis, grades 10–12, and sexually active. ^b^Did not undergo gynecological examination (*n* = 416), did not submit a questionnaire (*n* = 42), did not submit urine (*n* = 295), and 301 had overlapping reasons for being excluded.

A total of 933 participants from 32 schools were willing to be tested for STIs (Figure 1). One or more STIs were found in 819/930 (88.1%) of the participants and FGS was found in 210/933 (22.5%). More than half of these rural secondary school-going young women had been pregnant (Table 1). The mean age was 18.7 years (SD, 1.6), indicating that approximately half of them had missed a year or more of schooling. Mean number of lifetime partners was 1.9 (SD, 0.5), age at sexual debut 16.6 (SD, 1.5), and mean number of partners the last month was 0.7 (SD, 0.5). Only 25% (227/926) had used condoms the last week. Most women had been exposed to fresh water at high risk of schistosomiasis contamination at some point in their lives (881/930 [94.7%]). Urinary *S. haematobium* ova excretion was found in 256/840 (30.5%). In the positive cases, the geometric mean intensity urinary *S. haematobium* was 57.9 eggs per 10 mL. There was no difference in mean age of those who had FGS compared with those who did not. Evidence of a previous HSV infection (serology) was found in 286/900 (31.8%) of the participants. However, only a handful of pupils had current blisters. The prevalence of current *Chlamydia*, HPV, trichomoniasis, and gonorrhea were 218/827 (26.4%), 149/551 (27.0%), 163/839 (19.4%), and 102/827 (12.3%), respectively.

**TABLE 1:** The Main Characteristics of Participants with FGS

Variable	No. participants *n* (%)
Urinary schistosomiasis*^a^*	256/840 (30.5)
FGS manifestation^12,19^	
- Any sandy patches of cervix or vaginal wall	210/933 (22.5)
Superficial grainy sandy patches	84/933 (9.0)
Deep grainy sandy patches	51/933 (5.5)
Homogenous yellow sandy patches	109/933 (11.7)
- Rubbery papules	0/933 (0)
- Abnormal blood vessels	277/933 (29.7)
Lifetime risk water contact*^b^*	884/930 (94.7)
Religions and language	
Christian	744/927 (80.3)
Shembe*^c^*	152/927 (16.4)
Other/no religious affiliation	31/927 (3.3)
IsiZulu language spoken at home	923/933 (98.9)
Highest education level among family members (not participants)	
Tertiary*^d^* education	97/926 (10.4)
Secondary*^e^* education	827/926 (88.6)
Primary*^f^* school/no education	2/926 (0.2)
Obstetric history	
Previously pregnant	478/933 (51)
Parity, median (interquartile range)	1 (1–3)

Some points were not answered in the questionnaire.

*^a^Schistosoma haematobium* ova found in a single urine sample, some did not submit urine samples.

*^b^*River, dam, pond, or stream.

*^c^*Local popular religion.

*^d^*University level.

*^e^*8–12 years of school.

*^f^*1–7 years of school.

FGS indicates female genital schistosomiasis.

**FIGURE 2:**
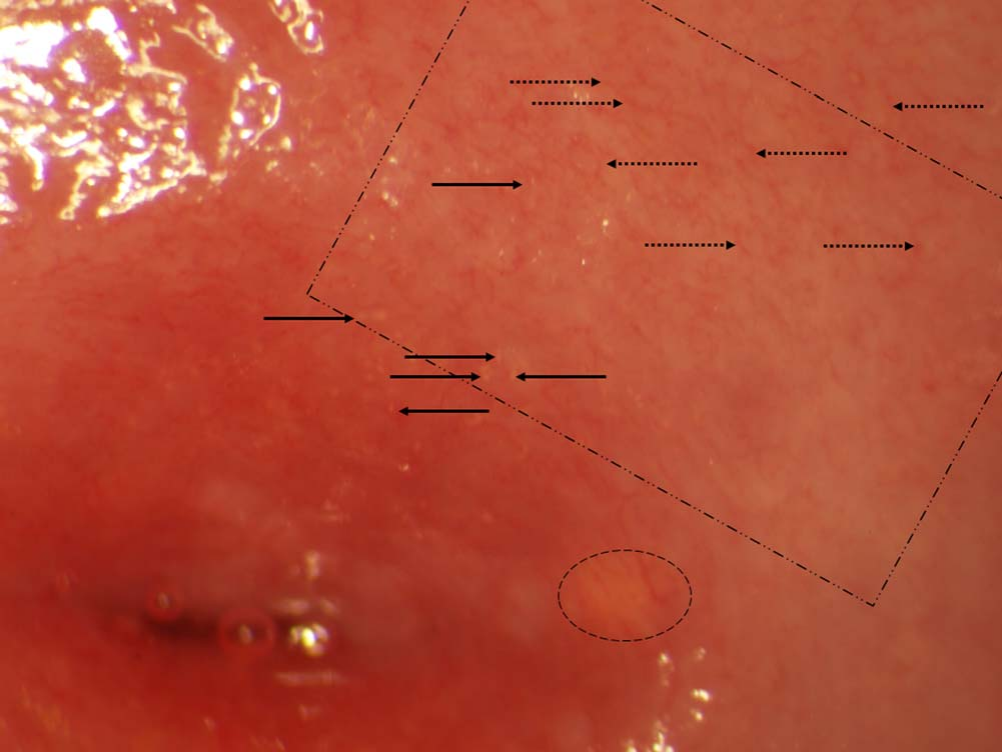
Lesions of FGS in a young South African woman. Solid arrows: Grainy sandy patch embedded in homogenous yellow patch (rectangle). Hatched arrows: Abnormal blood vessels. Oval: Nabothian cyst.

Human papillomavirus testing was done in a subsample of 551 patients (55%) and was positively associated with FGS. When HPV was analyzed against the subtypes of sandy patches seen in Figure 2, it was positively associated with superficial grainy sandy patches (age-adjusted odds ratio [OR] = 1.85; 95% CI = 1.05–3.245; *p* = .03) and homogenous yellow patches (adjusted OR [AOR] = 2.43; 95% CI = 1.49–4.0; *p* < .001). Human papillomavirus was however, not associated with deep grainy patches (age-adjusted OR = 0.70; 95% CI = 0.30–1.7; *p* = .42), but the sample size was small (51 cases).

There was significantly less *C. trachomatis* in the FGS-positive group than in the FGS-negative, after controlling for age, HSV serology, and trichomoniasis. *Chlamydia* was also negatively associated with superficial grainy sandy patches (age-adjusted OR = 0.44; 95% CI = 0.26–0.75; *p* = .002) and deep grainy patches (AOR = 0.30; 95% CI = 0.12–0.77; *p* = .01). Homogenous yellow patches showed the same tendency (AOR = 0.71; 95% CI = 0.42–1.19; *p* = .19). Similarly, the chlamydia prevalence tended to be lower in the urinary schistosomiasis-positive cases than in urinary schistosomiasis-negative, although this difference was not significant (AOR = 0.70; 95% CI = 0.45–1.02; *p* = .06). Half (109/218 [50.0%]) of the *C. trachomatis* cases were clustered in 8/32 (25.0%) schools, indicating possibly some super-spreader events. After stratification, taking out the chlamydia high-endemic schools, FGS remained negatively associated with *Chlamydia*, after adjusting for age, trichomoniasis, and HSV serology (AOR = 0.41; 95% CI = 0.22–0.79; *p* = .007), but not in the 8 schools, each of which had more than 35% *Chlamydia*. Of the study participants, 12.6% (110/876) had been treated for an STI previously. There was a significant association with having HSV (OR = 1.5; 95% CI = 1.2–1.9, *p* = .001) but no association was found with FGS (*p* = .97), *Chlamydia* (*p* = .33), dual infection with FGS and *Chlamydia* (0.45), or other STIs.

*Trichomonas vaginalis* was associated with the clinical finding of “homogenous yellow patches” (age-adjusted OR = 1.74; 95% CI = 1.10–2.75; *p* = .019). No other STIs were associated with grainy sandy patches (data not shown). Positive HSV serology and current trichomoniasis were higher in the FGS-positive group than in the FGS-negative but the differences were not significant. The mean bacterial vaginosis scores were the same in FGS-positive and negative populations. Only 29/901 (3.2%) cases were positive for *T. pallidum*, and there was no significant difference between the groups (*p* = .49).

As expected, FGS was positively associated with urinary schistosomiasis (OR = 2.32; 95% CI = 1.77–3.02; *p* < .001). Female genital schistosomiasis was found not only in 89/256 (34.8%) of those who were positive for urinary schistosomiasis but also in 110/584 (18.8%) of the participants where urinary schistosomiasis was not detected, showing that not all with urinary schistosomiasis have FGS and that urine analysis alone does not suffice for FGS detection. Among the 5 participants who denied any waterbody contact, none were diagnosed with urinary schistosomiasis, although sandy patches were found in 1 case. Urinary schistosomiasis was not associated with any of the other reproductive tract infections.

As further exploration, we included HPV in the multivariable analysis of Table 2. Human papillomavirus remained significantly associated with FGS (AOR = 1.71; 95% CI = 1.11–2.38; *p* = .016). There was less *Chlamydia* in the FGS-positive than in the FGS-negative, but the difference was not statistically significant (AOR = 0.67; 95% CI = 0.42–1.07; *p* = .096).

**TABLE 2:** The Association Between STIs and FGS in Young Women

	FGS+ *^a^*	FGS−	OR (95% CI)*^b^*	*p^c^*	AOR*^d^* (95% CI)	*p^e^*
Mean age, y (SD)	18.7 (1.6)	18.7 (1.6)		0.97		
HSV-2 serology*^f^*	36.9% (75/203)	30.4% (212/697)	1.39 (1.00–1.93)	0.079	1.36 (0.97–1.89)	0.077
*T. vaginalis* PCR*^g^*	24.2% (50/207)	18.1% (113/632)	1.44 (0.99–2.10)	0.055	1.44 (0.99–2.097)	0.056
*C. trachomatis^h^*	19.6% (37/189)	28.4% (181/638)	0.62 (0.41–0.92)	0.017	0.61 (0.41–0.92)	0.018
HPV	34.9% (52/149)	23.9% (96/402)	1.71 (1.14–2.56)	0.010	NI*^i^*	NI
*T. pallidum^j^*	4.0% (8/201)	3.0% (21/700)	1.34 (0.56–3.07)	0.49	NI*^k^*	NI
HSV PCR*^l^*	4.7% (5/107)	4.9% (12/244)	0.95 (0.33–2.76)	0.92	NI*^k^*	NI
*N. gonorrhea^h^*	13.8% (26/189)	11.9% (76/638)	1.18 (0.73–1.90)	0.50	NI	NI
BV*^m^*	61.7% (115/185)	62.2% (375/608)	1.01 (0.73–1.43)	0.91	NI	NI
Mean BV score (SD)	6.5 (2.8)	6.6 (2.5)
*Candida* species*^n^*	7.5% (14/186)	9.0% (55/608)	0.97 (0.74–1.27)	0.80	NI	NI
Mean candidiasis score (SD	0.2 (0.6)	0.2 (0.6)

Denominator varies because specimens were not available for all analyses.

*^a^*FGS determined by colposcopic inspection.

*^b^*Odds ratio (95% confidence interval).

*^c^*Chi-square test.

*^d^*Adjusted for age.

*^e^*Logistic regression.

*^f^*Herpes simplex virus.

*^g^*In-house PCR.

*^h^*Strand displacement assay.

*^i^*Not included (NI) in multivariable logistic regression analysis due to small sample size; however, explored in last paragraph of results.

*^j^*Rapid plasma reagin and *T. pallidum* hemagglutination assays.

*^k^*Not included in multivariable analysis due to *p* > .2.

*^l^*Only 351 analyzed (financial reasons).

*^m^*Nugent criteria, score > 7.

*^n^*Microscopy.

BV indicates bacterial vaginosis.

Comparing FGS-positive and negative, there was no difference in duration since the last sexual intercourse (*p* = .37), lifetime partners (*p* = .29), age at sexual debut (*p* = .34), number of partners the last month (*p* = .66), or condom use (*p* = .41).

## DISCUSSION

Female genital schistosomiasis was almost as common as *Chlamydia*, with both infections affecting more than one fifth of this young rural population. As published previously, the study shows that FGS seems to be a risk factor for HPV.^24,25^ Unexpectedly, the FGS-positive had less concurrent chlamydia infection compared with those without FGS. This has not been reported previously.

Female genital schistosomiasis manifestations cause a chronic state of intravaginal inflammation that could aid the acquisition of STIs.^9,26^ This inflammation may cause symptoms and may make women go to local health institutions for treatment. Diagnosis in rural, low-resource areas is almost invariably based on patient history and point-of-care inspection, very rarely confirmed by laboratory analyses.^27^ The *S. haematobium* transmission and likewise FGS are likely to have occurred in early childhood and therefore before STIs,^8^ indicating that there are intravaginal damages already from a young age. If the vaginal discharge has been constant since childhood, it could be argued that it would be interpreted as normal by the patients.^8,28^ However, 2 studies found that adults with FGS report having abnormal discharge.^29,30^ This may indicate that they experience changes in discharge, possibly because the bleeding and ulcerous surfaces of FGS are super-infected by other agents such as bacteria, as has been found for *S. haematobium* of the bladder in mouse models.^31^ Refractory symptoms must, however, be interpreted with caution, reinfection with STIs is common, symptoms may persist for a while after adequate treatment, or persisting symptoms may be caused by FGS.

There was an overall trend of higher prevalence of STIs in the FGS-positive participants. However, *Chlamydia* was the exception. *Chlamydia* is often asymptomatic.^32^ Young women with FGS and (asymptomatic) *Chlamydia* may have sought STI treatment (provided for free by the local clinics) more often than those without FGS. Alternatively, dual infection (with FGS and *Chlamydia*) might cause intensified symptoms, leading to health-seeking behavior and antibiotic treatment for vaginal discharge.^27^ This could not be confirmed in our study.

Our study's conclusions have the following limitations. To confirm the small differences between the groups, we would have had to have a larger sample size. Almost all participants in this population had local freshwater contact at some point in their lives, and they may have had FGS previously, diluting the findings of this study. The WHO currently recommends visual pelvic examination for diagnosis of FGS,^10^ whereas in areas where laboratory analyses are not available, WHO recommends only STI treatment based on patient information or genital examination for vaginal discharge, not mentioning FGS.^33^ Urine sampling cannot be used for FGS diagnosis.^2^ Due to logistic and resource constraints, only 1 urine sample was collected per participant. Multiple samples would likely have yielded a higher prevalence of urinary schistosomiasis due to day-to-day variations in egg excretion. This was a limitation in this study. Furthermore, FGS diagnosis is dependent on the colposcopic inspection of all intravaginal surfaces, including the fornices and posterior and anterior vaginal walls.^10^ Therefore, we may also have missed some FGS-positive cases, and we cannot preclude that STIs were sometimes misdiagnosed as FGS. This might have obscured differences between the FGS-positive and FGS-negative groups.^3^

The symptoms of FGS overlap with symptoms of the various STIs causing discharge, a burning sensation in the genitals, and ulcers.^8^ However, in countries where FGS is endemic, laboratory testing for STIs and examinations are often not performed. In rural Africa, abnormal discharge and genital ulcers are currently managed syndromically as STIs, without laboratory testing.^34^ The current syndromic management for vaginal discharge entails treatment for *Chlamydia*, gonorrhea, and trichomoniasis, but not for FGS.^34^ Therefore, patients may be inadvertently overtreated for STIs and undertreated/not treated for FGS.

Examinations for FGS rely on clinical expertise and are not yet performed in routine health care.^10,26^ The risk of misdiagnosis of FGS as an STI warrants rewriting the syndromic management protocols and algorithms for genital tract disease, especially in endemic areas. Furthermore, symptoms of STIs and FGS are also found in children with *S. haematobium.*^8^ Yet, health professionals are not taught about FGS, and FGS is not on the list of differential diagnoses for genital ulcers and discharge. Therefore, changes in the health professionals' curricula and the protocols for management of reproductive tract diseases in females are necessary.^35,36^ Antischistosomal treatment should be added to the treatment protocols and FGS should be considered among the differential diagnoses.

Preventive treatment for *S. haematobium* with praziquantel is not expensive and readily available and may provide a reduction in FGS occurrence. Further research is needed to understand the physiological mechanisms behind the sandy patches and to explore if praziquantel treatment can reduce the incidence of HPV and subsequent cervical cancers.
